# Volumetric Evaluation of Voids and Gaps of Different Calcium-Silicate Based Materials Used in Furcal Perforations: A Micro-CT Study

**DOI:** 10.3390/dj10030041

**Published:** 2022-03-09

**Authors:** Cassia Cestari Toia, Fabricio Batista Teixeira, Carolina Cucco, Marcia Carneiro Valera, Bruno Neves Cavalcanti

**Affiliations:** 1 Department of Operative Dentistry, Endodontics Division, Institute of Science and Technology, São Paulo State University (UNESP), Sao Jose dos Campos 12245-000, Brazil; cassia.cestari@outlook.com (C.C.T.); marcia.valera@unesp.br (M.C.V.); 2 Department of Endodontics, University of Iowa College of Dentistry and Dental Clinics, Iowa City, IA 52242, USA; fabricio-teixeira@uiowa.edu (F.B.T.); carolina-cucco@uiowa.edu (C.C.); 3 Department of Cariology, Restorative Sciences and Endodontics, University of Michigan School of Dentistry, Ann Arbor, MI 48109, USA

**Keywords:** furcal perforation, gaps, micro–computed tomography, voids

## Abstract

This study aimed at evaluating volumetrically gaps and voids of calcium-silicate based materials of different generations and handling properties (BC—Endosequence BC RRM-Fast Set Condensable Putty, MTA—ProRoot MTA, and BIO—Biodentine) in simulated furcal perforations in an ex vivo setup by microcomputed tomography (Micro-CT) analysis. Thirty-six extracted human mandibular molars with sound furcation areas were selected. Standardized perforations were created in the furcation area of the pulp chamber using #4 diamond burs. The specimens were randomly assigned to three groups (BC, MTA and BIO; *n* = 12). Samples were then scanned (SkyScan 1172; Bruker-microCT, Kontich, Belgium), and three-dimensional (3D) images reconstructed. The relative volume of gaps (VG%) and voids (VV%) present on each material was calculated. Data were analyzed using one-way analysis of variance (ANOVA) and Tukey’s HSD test (*p* < 0.05). Mean VG% for BC, MTA, and BIO groups were, respectively, 0.513%, 1.128%, 1.460%, with BC presenting statistically (*p* < 0.05) fewer gaps formation than the other groups. Mean VV% were, respectively, 0.018%, 0.037%, and 0.065%. The was no statistical difference regarding VV%. There were no gap-free and void-free samples. BC group had the lowest VG% among the groups with a significant statistical difference (*p* < 0.05).

## 1. Introduction

Furcal perforations are overall considered inconvenient accidents for clinicians. They happen due to several reasons, including extensive caries, resorptions, or iatrogenic factors [1] These incidents create a pathological path between the root canal system and the periodontium usually followed by microorganisms, inflammatory tissue, and bone loss, that may provide an adverse scenario, particularly when there is a radiolucent associated with the perforation site [2,3]. The prognosis is affected by several factors, such as exposure time, size, location [3,4], and also by the filling ability of the repair material in guaranteeing an hermetic seal [5,6]. This can be achieved by avoiding the development of gaps between the dentin walls and the material, and voids within the material content, minimizing bacterial microleakage, thus enhancing the success rates of the sealing of furcal perforations [6] and the perforation walls. Over the years, a wide range of materials has been applied for furcal perforation repair such as zinc-oxide eugenol, glass ionomer, and resin-based cement [7,8,9]. However, bioactive materials, also known as calcium-silicate based materials, strongly came into clinical practice since they reduce the likelihood of tooth loss by enhancing the healing outcomes [10]. These materials promote not only a tight seal by the formation of an interfacial layer between the material and root surfaces, but also lead the regeneration of periodontal and bone tissues through the release of calcium ions, and the production of calcium hydroxide and apatite crystals [11,12].

MTA was first introduced in the 90s [13] and since then, has been advocated as a gold standard material for perforation repair and other implications due to its favorable properties [9,11]. However, MTA presents some problematic handling characteristics, poor radiopacity, and long setting time [14,15,16]. Therefore, several calcium silicate materials have been developed to beat the disadvantages of MTA. As an alternative to the MTA, Biodentine (Septodont, Saint Maur des Fosse’s, France) became commercially available in 2009 as a second-generation calcium silicate-based material. The setting time, which has been reported by the manufacturer to be 12 min, is shortened due to some of its components, such as calcium carbonate and the water-based solution, containing calcium chloride and carboxylate in the liquid [17]. More recently, putty-like repair materials such as Endosequence BC RRM-Fast Set Condensable Putty (Brasseler USA, Savannah, GA, USA) were introduced as third-generation calcium silicate-based materials. These materials may provide an easy and swift application since no mixing is required previous to its placement in the furcal perforation [10,18]. Putties have shown good outcomes in cases of root resorptions, vital pulp therapies, and microsurgeries [19].

The evaluation of calcium silicate-based materials in furcal perforation repair is still limited since valuable alternatives for this clinical condition are mostly based on unpredictable case reports. Those investigations included unclear features that may have affected the outcome of perforation repair other than the investigated materials themselves increasing the need for studies under controlled conditions. Regarding this, microcomputed tomography (Micro-CT) analysis is an alternative method to assess the physical behavior of biomaterials [20], providing a precise assessment of the samples without the tooth destruction [21,22] being used for the assessment of 3D microstructures in ex vivo models [23]. Micro-CT holds an essential role in the image analyses because of its capacity in differentiating the dentinal wall from the filling materials, gaps and voids using different grayscale thresholds [24]. This tool also contributes to simulating the clinical practice, increasing the quality and predictability of the endodontic treatment [25,26,27].

Considering the lack of studies evaluating gaps and voids in cases of furcal perforation, and the advantages of Micro-CT analysis and its ability to complement conventional tests for endodontic materials, the present study aimed at evaluating the volume of gaps and voids of calcium silicate-based materials with different handling and mixing features in first mandibular molars with furcal perforations.

## 2. Materials and Methods

### 2.1. Sample Size Estimation

The local institutional review board (Institutional Review Boards of the University of Iowa, 27 March 2019) approved the use of freshly extracted human teeth in the present study under registration no. 201903881. Due to the absence of specific studies using the Micro-CT analysis to evaluate gaps and voids in furcal perforations filled by different materials, a pilot study was performed by the same operator (C.C.T.), a specialist with 5 years of experience, who was also responsible for the experimental procedure and evaluation. Based on the data from the pilot study regarding percentage values of gaps and voids, the sample size calculation revealed that a minimum of eight teeth per group would be appropriate to show a 5% difference in the volume percentage of gaps and voids with a power of 90%.

### 2.2. Sample Selection

Thirty-six (*n* = 12 per group) freshly extracted human first mandibular molars with sound furcation area were selected based on general dimensions and a similar pulp chamber height of at least 2 mm and less than 3 m. By evaluating teeth under a dental microscope (OPMI PICO; Carl Zeiss, Goettingen, Germany) at 10× magnification, teeth presenting fractures, cracks, perforations, caries, resorption and previous root canal treatment were excluded. Calculus bone and soft tissue were removed with Gracey curettes. To avoid bacteria proliferation, teeth were stored in 0.1% thymol solution at 4 °C prior to the study enrollment.

### 2.3. Experimental Procedure

**Figure 1:**
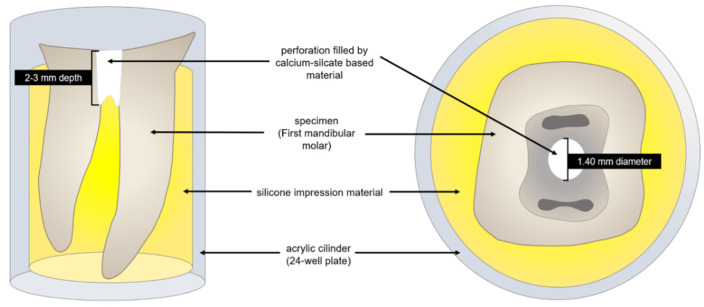
Experimental set-up. Specimen placed individually sectioned in acrylic cylinders of 24-well plates (Sigma-Aldrich St. Louis, MO, USA) with silicone impression material (EXAFLEX^®^ Vinyl Polysiloxane Impression Material, Putty Standard Package—GC America, Inc., Alsip, IL, USA) to mimic the alveolar bone and periodontal ligament.

The occlusal and middle thirds of the crowns were horizontally cross-sectioned and removed with a carborundum disc and then discarded, to guarantee the exposure of the furcal region. Pulp tissue was removed with the aid of dentin curettes and teeth cleaned. Cavities simulating furcal perforations were created in the middle of the pulp chamber floor by using diamond burs to their full depth in a high-speed handpiece under water cooling. The widths of all perforations were standardized to the diameter (1.40 mm) of #4 diamond burs and the depth of the perforation ranged from 2 to 3 mm according to the dentin-cementum thickness in the furcation area. All the procedures were performed (by C.C.T.) under a dental microscope (OPMI PICO; Carl Zeiss, Goettingen, Germany) at 10× magnification. Prior to the calcium silicate-based materials placement, all cavities were carefully rinsed with saline solution, dried with paper points (Dentsply Maillefer, Ballaigues, Switzerland), and filled with cotton pellets. Teeth were then placed individually in a 24-well plate (Sigma-Aldrich St. Louis, MO, USA) with silicone impression material (EXAFLEX^®^ Vinyl Polysiloxane Impression Material, Putty Standard Package—GC America, Inc., Alsip, IL, USA) to mimic the alveolar bone and periodontal ligament, avoiding the extrusion of the materials during their placement (Figure 1).

After the impression material polymerization, the cotton pellets were then removed and the cavities carefully inspected before the filling procedure.

Specimens were randomly assigned into three groups as follows:

1. BC—Endosequence BC RRM-Fast Set Condensable Putty (BC RRM-FS; Brasseler USA, Savannah, GA, USA);
2. MTA—ProRoot MTA (PRM; Dentsply Tulsa Dental, Tulsa, OK, USA), and
3. BIO—Biodentine (Biodentine Active Biosilicate Technology Scientific File, Septodont, Paris, France).

Both MTA and BIO were mixed according to the instructions provided by the manufacturer. For the BC group, no mixing was needed. Cavities were incrementally filled with the perforation repair materials to their extension by the same single operator in order of one by one. Each increment was gently compacted by using a hand plugger (Buchanan Hand Plugger, size no. 1, red tip; SybronEndo, Orange, CA, USA). For the MTA group, the material was also carried by the microapical placement (MAP) system, as specified by the manufacturer. As calcium silicate materials need moisture to set, teeth were stored at 37 °C and relative humidity for one week, and afterward subjected to Micro-CT scanning.

### 2.4. Calibration of Evaluator and of the Evaluations

The same operator (C.C.T.) who performed the pilot study and the experimental procedure also evaluated the samples. To guarantee a standardization, and an accurate assessment of the data, a calibration of the density ranges of each material, dentinal walls and empty spaces (gaps and voids) was performed by analyzing successive Micro-CT images of each tooth. In addition, the Intraclass Correlation Coefficient (ICC) was calculated (Mangold International Gmbh, Arnstorf, Germany, 2015) to assess the intra-rater reliability during the analysis for both the pilot and final study. The intra-rater reliability score was 1.0, an excellent reproducibility.

### 2.5. Micro-CT Evaluation

**Figure 2:**
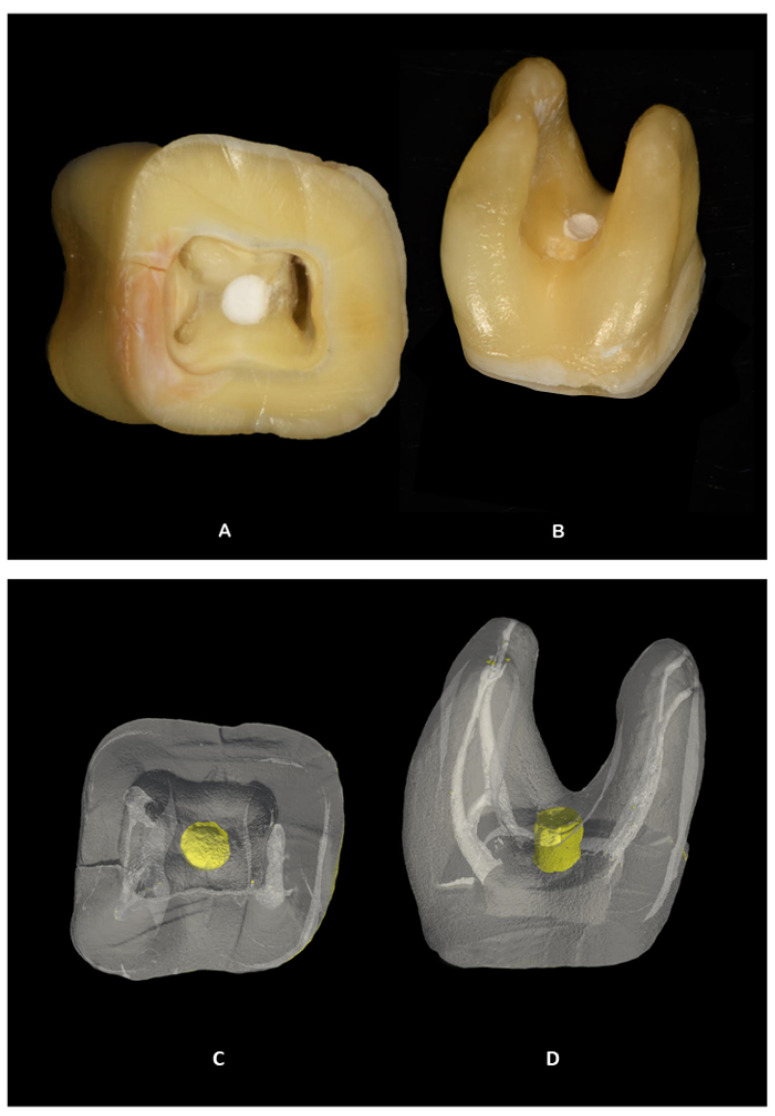
Pulp chamber (**A**) and root (**B**) views of the standardized cavity simulating a furcal perforation filled with one calcium silicate-based material. Pulp chamber (**C**) and root/furcal (**D**) views of the three-dimensional (3D) reconstruction of the same tooth using ParaView (v5.8.1, https://www.paraview.org/, accessed on 4 March 2022). Yellow represents the material.

After the specimen incubation in relative humidity, teeth were scanned using an ex vivo Micro-CT scanner (SkyScan 1172; Bruker-microCT, Kontich, Belgium) to analyze the volume of gaps (unfilled areas between the surrounding dentin and the perforation repair material) and voids (unfilled areas within the material) in cavities simulating furcal perforation. Samples were scanned at 125 kV and 80 μA using a voxel size of 21 (μm), a 1-mm thick Aluminium filter, a rotation step of 0.6 (deg), and an exposure time of 2630 s. Prior to each scan, a flat field correction was performed. Digital data were reconstructed using NRecon 1.7.1.0 software (Bruker Micro-CT, SkyScan, Belgium). Reconstructed images were first visualized by Paraview (v5.8.1., https://www.paraview.org/, accessed on 4 March 2022) (Figure 2) and then inspected and reoriented by DataViewer 1.5.2.4 (Bruker Micro-CT, SkyScan, Belgium) to achieve standardization among the samples.

### 2.6. Volume Calculation

**Figure 3:**
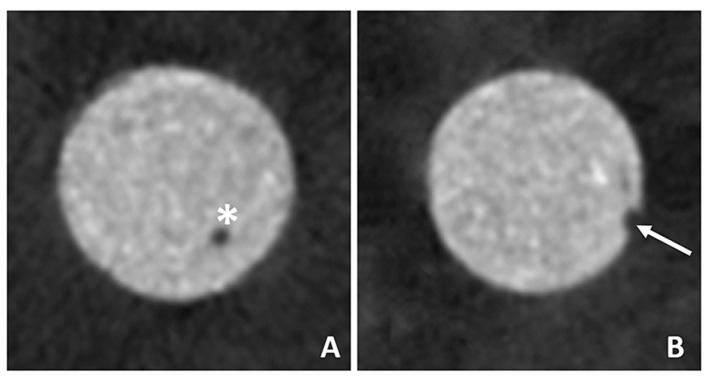
Cross sections of the cavities filled with the calcium silicate-based material. (**A**): Asterisk (*) represents voids within the material and (**B**): arrow (→) represents gaps between the dentin wall and the material.

The volumetric data (mm^3^) of the calcium-silicate based materials within the cavities (Vm), gaps (Vg) and voids (Vv) were obtained by using the 3D (three-dimensional) analysis tool of the CT-An software (version 1.16.4.1, Bruker Micro-CT, SkyScan, Belgium). The region of interest (ROI) was selected in an area over the furcal perforation depth by using the “top” and “bottom” tools. Once a suitable ROI was selected, the proper grayscale threshold was established to distinguish the root dentin wall from the cavity, comprehending the repair material itself, gaps, and voids (Figure 3). The volume of interest (VOI) was obtained after the delimitation of the cavity (Vc) according to its margins. A density from 80 to 255 was assigned to be the volume of the material (Vm) and a density range from 0 to 80 was used to the calculate the volume of voids (Vv) of each sample according to the pilot study. The volume of the perforation repair material (Vm) and voids (Vv) were then subtracted from the volume of the furcation cavity (Vc), to assess the volume of gaps (Vg). The final volume percentage of gaps (VG%) and voids (VV%) was calculated using the following formulas:

VG% = Vg/(Vg + Vm) × 100, and

VV% = Vv/(Vv + Vm) × 100.

### 2.7. Statistical Analysis

Data presented normal distribution. The percentages (%) of gaps and voids were compared between the groups by using one-way analysis of variance (ANOVA) and Tukey’s HSD as post-hoc test. Statistical analysis was performed using Microsoft Excel (v. 15.5, Redmond, WA, USA). The level of significance adopted was 5%.

## 3. Results

**Figure 4:**
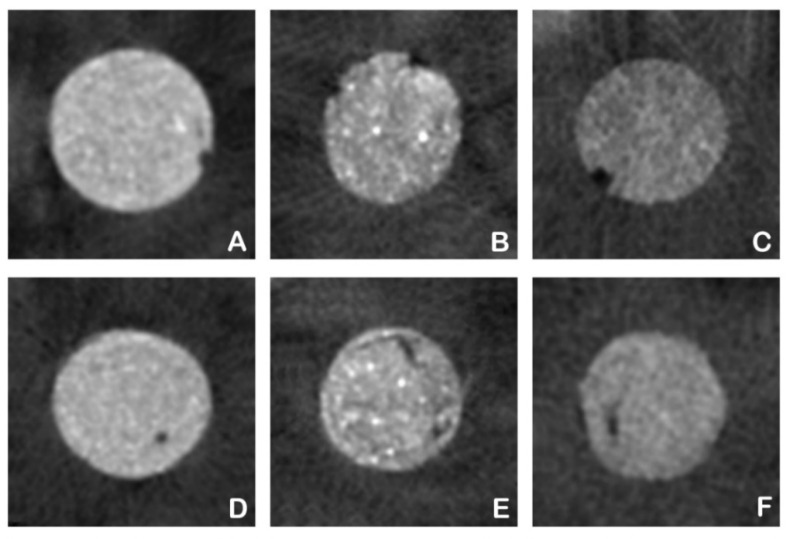
Representative cross-sectional Micro-CT images of the cavities simulating the furcal perforation filled by three different calcium silicate-based materials. Gaps are shown on: (**A**) for BC, (**B**) for MTA and (**C**) for BIO groups and voids are shown on: (**D**) for BC, (**E**) for MTA and (**F**) for BIO groups.

Micro-CT analysis exposed a clear overview of tooth dentin, calcium silicate-based materials, and unfilled areas at assigned grey values. All samples showed both gaps and voids after Micro-CT analysis (Figure 4).

Microcomputed tomography showed that the BC group had the lowest VG% among the studied materials (*p* < 0.05) and no significant statistical difference was found regarding VV% (Table 1).

## 4. Discussion

Over the years, tooth extraction was the treatment of choice in cases involving the furcation area. However, the furcal perforation repair is the best option in terms of dental structure preservation. The evaluation of the furcal perforation healing is still limited once valuable alternatives for this clinical condition are frequently based on unreliable clinical reports, increasing the need for more studies under controlled conditions [28] Even with an increasing tendency towards the application of calcium silicate-based materials in Endodontics [29], there are a small number of studies concerning their sealing ability in furcal perforation repair through Micro-CT. Concerning this, the filling ability of calcium silicate-based materials, with different handling and mixing features, for the treatment of furcal perforation was evaluated in the current study by assessing the volume (mm^3^) percentage of gaps (VG%) and voids (VV%) through Micro-CT analysis.

High-resolution Micro-CT analysis is a highly accurate method which has been used for the assessment of 3D microstructures in ex vivo models [23]. Micro-CT holds an essential role in the image analyses because of its capacity in differentiating the dentinal wall from the furcal repair materials, and empty spaces by means of different grayscale thresholds [24]. Prior to the image acquisition, during the pilot study, the standardization of the thresholding from the calcium silicate-based materials and the empty spaces were obtained, ensuring an accurate assessment of the data. Due to the different radiopacity of the materials, density ranges were established in a wide range (varying from 80 to 255), avoiding a possible tendency in the final results. The region of interest (ROI) and the volume of interest (VOI) were defined by a length from 2 to 3 mm (furcal perforation depth) to hold samples standardized. Finally, the results were submitted to the Intraclass Correlation Coefficient (ICC) and an excellent agreement (1.0, Mangold International GmbH, 2015) among the data was found. Since ICC reflects in both degree of correlation and agreement between measurements, this study could be performed by a single operator, who also analyzed successive Micro-CT images of each tooth, taking into consideration the calibration of the density ranges from the calcium silicate-based materials, dentinal walls and the empty spaces (gaps and voids).

Unfilled areas should be prevented for a better prognosis since they act as a pathway for bacteria and their toxins through the perforation cavity [4]. For this reason, an efficient seal ought to avoid the progress of bacteria and their by-products from the pulp chamber to the surrounding periodontal tissues. Empty areas commonly originate from the air trapped in the material mass during its preparation or placement [30]. These deficiencies can also be related to the density and/or flow intrinsic to the material itself [30].

Willing to assess the filling capacity of calcium-silicate based materials with different generations, and handling/mixture characteristics, a hand-mixed material (Pro-Root MTA), a mechanically mixed one (Biodentine) and a ready-to-use putty (Endosequence BC RRM-Fast Set Condensable Putty) were selected. When the distribution of unfilled areas was evaluated, all the specimens presented both unfilled areas between the material and the surrounding dentin (gaps) and within the material (voids).

Based on our results, all samples showed a minor number of empty spaces represented by gaps and voids, with means varying from 0.513%, 1.128% to 1.460% for gaps, and from 0.018%, 0.037% to 0.065% for voids on BC, MTA, and BIO, respectively. Even though gaps and voids seem to be consistent structural features, while voids (VV%) were equally found among the groups (*p* > 0.05), BC showed fewer gaps (VG%) formation than the other groups (*p* < 0.05), as shown in Table 1. The premixed form in which Endosequence BC RRM-Fast Set Condensable Putty is commercialized may reduce the air entrapment in the mix, providing better adaptability to dentinal walls [19]. In addition, the distribution of the material within the cavity by “hydraulic condensation pressure” may improve its overall sealing and adaptation, since BC does not shrink but expands to a certain degree, being insoluble in tissue fluids [31]. Both MTA and BIO groups presented the same VG% with no statistical difference, unlike in a previous study in which Biodentine had less gap formation compared to MTA [32]. The use of the microapical placement (MAP) system for the MTA group in this study most likely enhanced its poor handling characteristics.

Building an ex vivo furcal perforation model is challenging because of the difficulty in simulating the anatomical relationship between a furcal cavity and the subjacent periapical tissues. For this reason, the use of a silicone impression material to mimic the alveolar bone and periodontal ligament avoiding the material extrusion was required. To overcome some limitations during the simulation, all cavities selected had a variation in their depth according to the dentin-cementum thickness in the furcation area (from 2 to 3 mm of height) and their widths were standardized to the diameter of the burs (1.40 mm). The final VG% and VV% were calculated, considering the total amount of the materials (Vm) to overcome these limitations.

As a promising technique, the Micro-CT analysis used in this study provided a great understanding of the volumetric measurements of gaps and voids because of its highly accurate features, since two dimensional techniques cannot be suitable to measure a three-dimensional structure [21,33]. To the best of our knowledge, this study is one of the first using Micro-CT to measure the percentage volume of gaps and voids in calcium silicate-based materials for furcal perforation repair. The present study verified Endosequence BC RRM-Fast Set Condensable Putty as a promising furcation repair material, due not only to its premixed form and handling properties, but also to its favorable sealing ability regarding its lower percentage of gaps (VG%).

## 5. Conclusions

None of tested materials were gaps and voids free. The Endosequence BC putty had the lowest volume of gaps among the three tested materials.

## Figures and Tables

**Table 1:** Average ± standard deviation of the percentage volumes of gaps (VG%) and voids (VV%) of the calcium-silicate based materials for perforation repair. * Indicates statistical difference (*p* < 0.05) from the other experimental groups.

Groups	Gaps (%)	Voids (%)
BC	0.513 ± 0.320 *	0.018 ± 0.030
MTA	1.128 ± 0.904	0.037 ± 0.086
BIO	1.460 ± 0.933	0.065 ± 0.075

## Data Availability

The data presented in this study are available on request from the corresponding author. The data are not publicly available due to multiple sources of financial support.
